# A Foley catheter ‘the jack of all trades’: a literature review of its common and novel uses

**DOI:** 10.1308/rcsann.2023.0003

**Published:** 2023-07-12

**Authors:** R Karmarkar, S Bodapati, L Yao, S Aroori

**Affiliations:** University Hospitals Plymouth NHS Trust, UK

**Keywords:** Foley catheter

## Abstract

The Foley catheter is one of the most commonly used devices in modern surgical practice. Developed for draining the urinary bladder, this humble catheter has been employed for many other purposes ranging from urine output monitoring to complex urological investigations. Over time, it has evolved into being applied in more complex and innovative ways in various other specialties apart from urology. In this review article, we describe some of the common and novel uses of this deceptively simple device, and discuss the scope of its application in modern medicine.

## Introduction

The Foley urinary catheter is one of the most frequently used devices in modern surgical practice although it is often taken for granted. In this review article, we present a detailed overview of this catheter, covering its origin and history, structure, licensed uses and other common uses for which it has been deemed appropriate. We also describe some novel applications of the Foley catheter as reported in the literature and in our own practice. The many applications of this seemingly simple device make it worthy of a thorough study.

## History

The word catheter is derived from the ancient Greek *kathiénai*, which means “to thrust into” or “to send down”.^1^ The first references to the use of a catheter-like system can be found in the ancient Indian texts of the *Sushruta Samhita*.^2^ Similar references are also seen in Greek and Chinese literature dating back to the first and second centuries BC, which describe the catheter as a hollow metal tube made of gold, silver, iron or wood and clarified butter used as a lubricant.^3,4^

In modern history, the catheter saw fundamental design changes with the introduction of a bend in the tip (“coude tip”) and the double bend (“bicoude tip”) for ease of insertion.^5^ With the advent of flexible materials in later years, the hollow metal tube was replaced with rubber. However, a vital problem remained unsolved: how to anchor the catheter. Anchorage was originally gained by taping, tying or sewing the catheter to the leg, which was cumbersome and painful for the patient. This issue was resolved when Frederick Foley introduced the self-retaining balloon anchorage system in 1935.^6^ It is still known as the Foley catheter to this day.

Since then, many variations have been made to the Foley catheter, including changes in materials, size and structure, allowing greater patient comfort and fewer complications. One of the more recent modifications includes the development of a third conduit so that the catheter can be used for continuous bladder irrigation. Yet another development involves impregnating the catheter with an antibacterial/silver film to reduce rates of bacteriuria and sepsis.^7^ While the humble catheter has progressed from a hollow metal tube to its current concept and variants, development continues further, especially with the use of urinary catheters as a long-term measure.

## Catheter features: structure, size and materials

**Figure 1:**
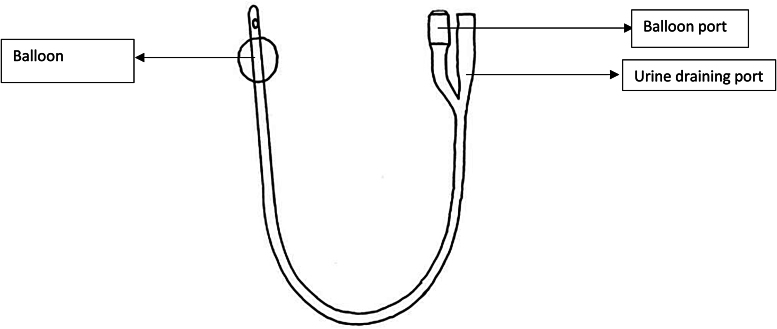
Typical structure of urinary catheter

Figure 1 shows the typical structure of a urinary catheter. The modern-day Foley catheter has two channels: a drainage channel (for urinary drainage) and an inflation channel (for inflating the balloon at the tip of the catheter to retain the catheter in the bladder). The smooth, rounded tip of the catheter extends beyond the balloon and one or more eyeholes are cut in the tube adjacent to the tip to allow urine to drain.^6,8^

Catheter size is usually expressed in French gauge (Fr). It is standard practice to choose the smallest catheter compatible with good drainage; 12–16Fr is usually adequate and only rarely is a catheter larger than 18Fr necessary.^6,8^ Urinary catheters are available in a variety of base materials as different materials provide different properties and are suitable for various purposes. The most common materials include polyvinyl chloride (PVC)/plastics. Catheters made of PVC tend to be stiff and uncomfortable for patients. For this reason, they are used for short periods only. Latex catheters are generally no longer in use owing to the possibility of life threatening anaphylaxis in patients with a latex allergy.^8^ Polytetrafluoroethylene coated catheters offer longer life and can be left in situ for up to 28 days. One hundred per cent silicone catheters are hypoallergenic and last 12 weeks.

## Traditional uses of the urinary Foley catheter

The various uses of the Foley catheter can be divided into two categories: traditional (for urinary drainage/urological investigations) and non-traditional (for non-urological specialties). As the name suggests, a urinary Foley catheter is used for urine drainage (both short-term and long-term). Table 1 gives a brief overview of its uses as a catheter in urology.

**Table 1:** Indications for urethral catheterisation^8^

Drainage	• Acute and chronic urinary retention • Incomplete bladder emptying • Following urological surgery (e.g. transurethral resection of bladder tumour)
Monitoring	• Monitoring fluid balance in critically ill patients
Instillation	• Instillation of drugs into the bladder • Obtaining urine samples
Investigations	• Radiological evaluation of the lower urinary tract (e.g. cystography/urethrography)
Palliative	• Intractable urinary incontinence • Protect against pressure sores • In terminally ill patients

## Other applications of the Foley catheter

In addition to its traditional uses, the Foley catheter has been employed in many other clinical settings. We have put together a non-exhaustive list of the various non-urological applications of Foley catheters below.

### Gastrointestinal surgery

#### As an alternative to gastrostomy/jejunostomy tube^9,10^

In the literature, Foley catheters have been reported to be frequently employed instead of a gastrostomy or jejunostomy tube for enteral feeding. The Foley catheter works well as a replacement for traditional gastrostomy tubes for three reasons: it is readily available, less expensive and easy to insert. (Nurses can change it in a nurse-led clinic.) However, this alternative use also comes with complications such as tube migration and intestinal obstruction due to the Foley balloon. Another downside is that a Foley catheter requires more frequent changes than traditional gastrostomy tubes. Lastly, using a urinary catheter as an enteral feeding tube has important implications for practice given that it is not licensed for this type of use, and in terms of consent, ethics and professional responsibilities.

#### Administration of enemas through a stoma^11^

**Figure 2:**
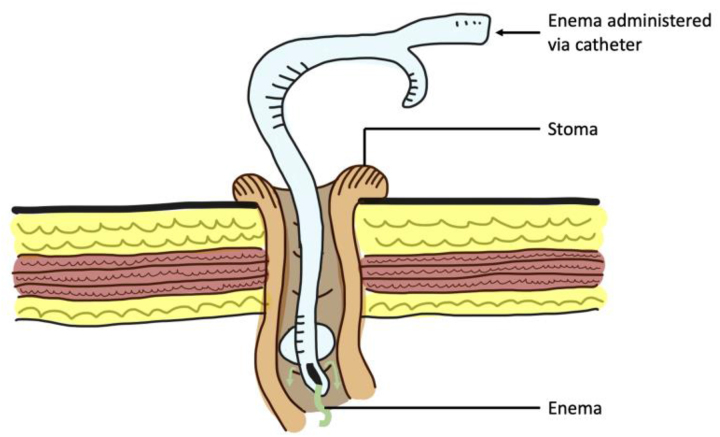
Use of catheter to administer enemas

Constipation is a common consequence in patients with a stoma. Administration of an enema through the stoma can be challenging as there is no sphincter activity around the stoma, resulting in spillage during administration and ineffective occlusion of the lumen after administration. A Foley catheter can be used to administer the enema through the stoma, overcoming these two challenges. The balloon fits snugly against the parietal wall and prevents seepage of the administered enema (Figure 2).

#### Paediatric intussusception^12–15^

A Foley catheter can be employed for hydrostatic or pneumatic reduction of paediatric intussusception. Intussusception in infants could be a life threatening condition and various non-operative techniques have been described for its treatment. Of the many methods considered, the use of a Foley catheter is an important one. The technique involves instillation of water or air via a Foley catheter inserted into the child's rectum with continuous pressure monitoring and ultrasonography to confirm resolution of the obstruction.

#### Duodenal stump leak^16–19^

**Figure 3:**
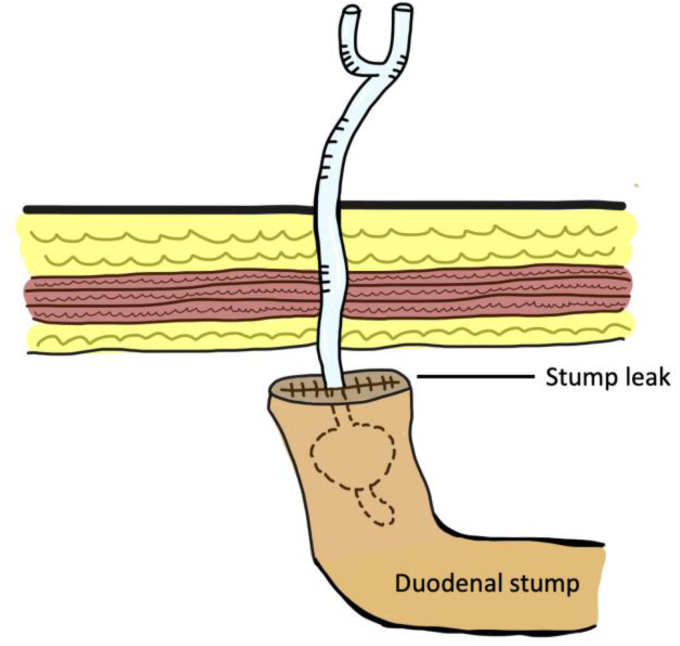
Management of duodenal stump leak

Duodenal stump blowout following gastrectomy has serious implications in terms of delaying enteral nutrition, prolonged hospital stay and delayed patient recovery. Interventional radiology guided percutaneous Foley catheter placement in the duodenal stump can overcome all of these issues and can prevent reoperation. Enteral feeding can be started early with the Foley balloon plugging the stump leak, thereby aiding recovery (Figure 3).

#### Oesophageal foreign bodies^20,21^

**Figure 4:**
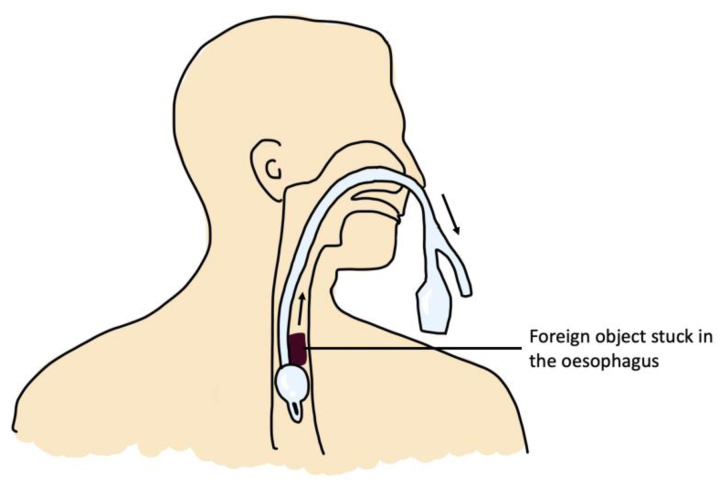
Removal of oesophageal foreign bodies

Foley catheters have been used to facilitate removal of oesophageal foreign bodies in the absence of oesophageal oedema or tracheal compromise. The catheter is inserted through the mouth under fluoroscopy guidance (Figure 4). Once the tip is distal to the foreign body, the Foley balloon is inflated and the catheter withdrawn while removing the foreign body. This method has significant advantages compared with endoscopic techniques in terms of cost, speed and avoiding anaesthesia.

### Hepatopancreatobiliary surgery

#### Cholecystostomy^22,23^

**Figure 5:**
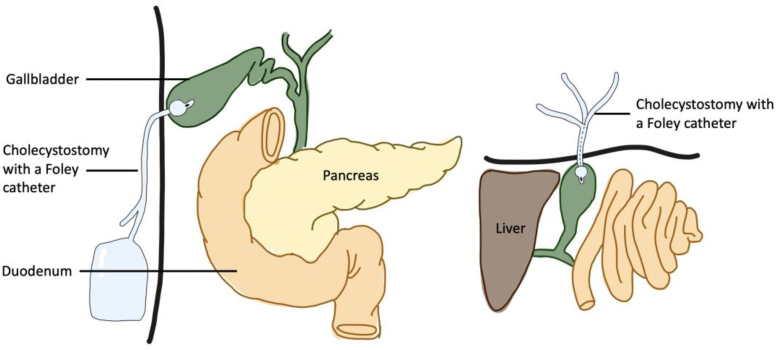
Foley catheter application in cholecystostomy

The Foley catheter has been used as a temporising measure for gallbladder drainage in acutely inflamed gallbladders where it was deemed inappropriate to proceed with a cholecystectomy via a laparoscopic approach (Figure 5). In one representative instance, the catheter was left in situ for 4–6 weeks until the inflammation settled and a delayed cholecystectomy was performed. Cholecystostomy with a Foley catheter can be employed as an alternative to conversion to open surgery.

#### Hepatic outflow obstruction^24,25^

Early hepatic venous outflow obstruction is a rare but severe complication of liver transplantation. The causes can be attributed mainly to technical problems such as malposition of the graft or twisting of the hepatic veins. Ectopic placement of a Foley catheter between the diaphragm and the graft has been reported to prevent kinking of the hepatic veins, thereby maintaining adequate outflow.

#### Sterilisation of liver hydatid cyst^26^

**Figure 6:**
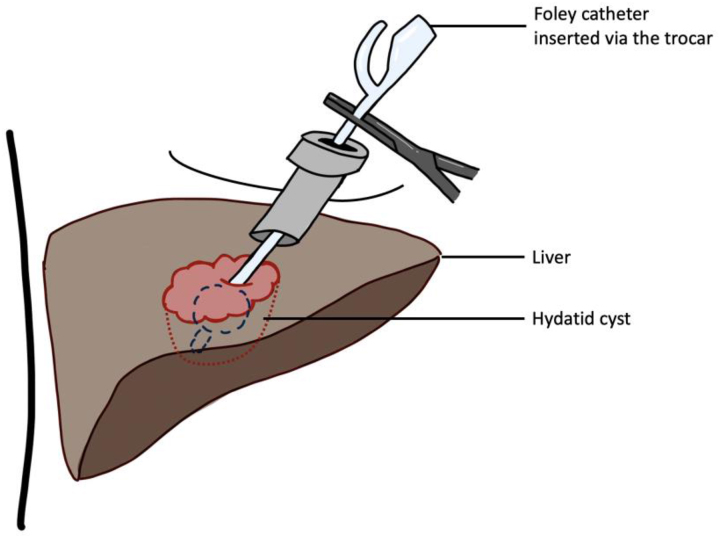
Sterilisation of liver hydatid cyst

Sterilisation of the cyst cavity is essential in the surgical management of hydatid cysts. A Foley catheter has been used in both the open technique and laparoscopic sterilisation of these cysts. The catheter is inserted in the cyst cavity and the Foley balloon is inflated (Figure 6). This is followed by injection of a scolicidal agent in the cyst cavity and all the fluid being aspirated a few minutes later. With this procedure, multiple cysts in different locations in the liver can be accessed.

#### Pringle manoeuvre in open and minimally invasive liver surgery^27^

The Pringle manoeuvre assists with the control of bleeding by halting the bleeding and allowing the surgeon to find the source. It is a crucial step during complex open and minimally invasive liver surgery. Traditionally, wet nylon tape passed through a suction tube has been used for this purpose (Rumel tourniquet technique). Recently, however, many centres have started using a Foley catheter when performing the Pringle manoeuvre, with resounding success.

**Figure 7:**
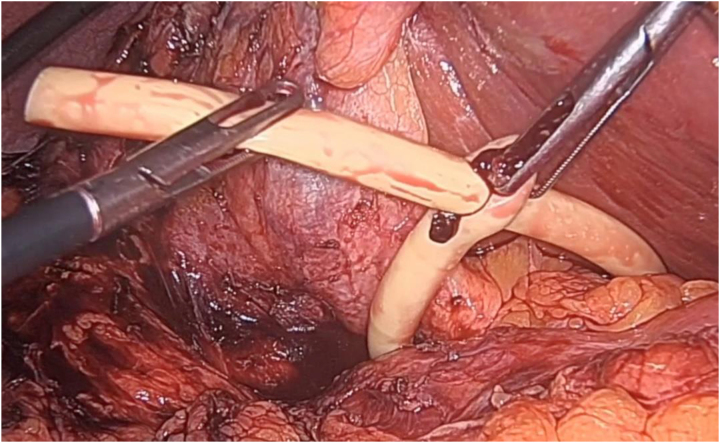
Pringle manoeuvre

The senior author now routinely undertakes the Pringle manoeuvre with a 14–16Fr Foley catheter (Figure 7). It has proved to be quicker and easier to use, and has a shorter learning curve. The blunt tip and flexible nature of the catheter allows effortless insertion into the foramen of Winslow. The surgeon does not need to insert any instruments to grab the nylon tape and so there is no risk of damaging the structures in the hepatoduodenal ligament. The Foley method is cheap as well as easy to teach and learn. There is also no requirement for an extra port during minimally invasive liver surgery as the Foley technique is intracorporeal.

### Haemorrhage control

#### Port site bleeding^28,29^

Port site bleeding following laparoscopic surgery is a common occurrence. It is a particular challenge for surgeons in the case of obese patients as the bleeding points are deeply situated, making it difficult to control them with deep sutures or electrocautery. The Foley catheter has proved to be an effective alternative to these methods for such patients. The catheter is introduced through the port site. The Foley balloon is then inflated to maximum capacity, ensuring complete arrest of bleeding in the peritoneal cavity, and removed after 24 hours.

#### Bleeding following trauma^30–34^

Several articles in the literature suggest that Foley balloon tamponade is a frequently employed technique for various types of traumatic bleeding. It is mainly helpful in cases of inaccessible bleeding sites such as deep penetrating neck injuries, deep solid organ injuries (e.g. parenchymal liver injuries) and cardiac injuries. It can be a temporary bridge to definitive control of bleeding in the operating theatre.

#### Vacuum assisted breast biopsy^35^

**Figure 8:**
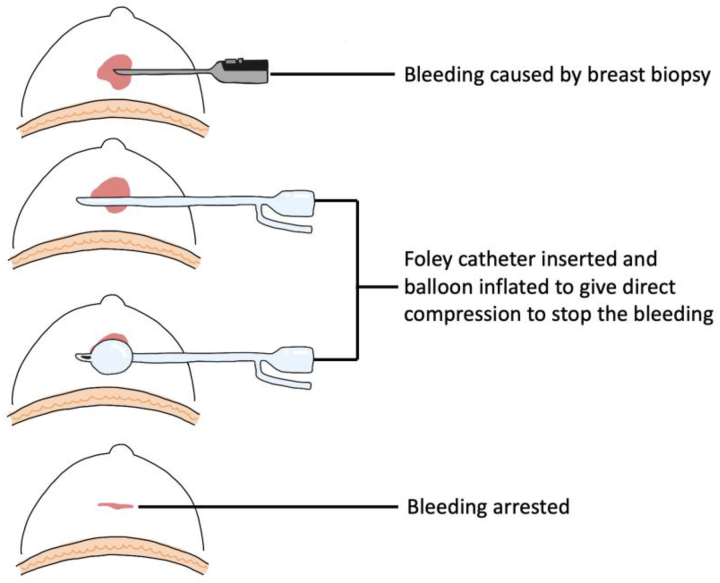
Control of bleeding following vacuum assisted breast biopsy

Interventional bleeding and post-interventional haematoma are the two most common complications of vacuum assisted breast biopsy. The same principle of Foley balloon tamponade has been used to control bleeding following breast biopsy (Figure 8), and has been reported as a safe and effective method of haemostasis.

#### Epistaxis^36–38^

Posterior nasal packing using Foley catheter tamponade has been reported as a safe, effective and rapid non-surgical means of controlling refractory posterior epistaxis. Its advantages include that no anaesthetist or theatre space is required for insertion.

### Obstetrics and gynaecology

#### Induction of labour^39,40^

The rates of induction of labour have been increasing worldwide. Cervical ripening is the first step in labour induction. The Foley catheter has been employed successfully as a mechanical method of cervical ripening. It has been compared with other pharmacological methods (such as intravaginal prostaglandins), and is considered equally safe and effective.

#### Hymen repair^41^

Use of a Foley catheter has been reported for imperforate hymen repair. A circular incision is made in the hymen, the catheter is inserted through the incision and the Foley balloon is inflated. This is followed by application of oestrogen cream locally to promote epithelialisation. The catheter is removed after 14 days. This has proved to be an easy yet effective alternative to other surgical procedures for imperforate hymen repair.

#### Bleeding during treatment for cervical and Caesarean scar pregnancy^42^

Foley catheters have been used either prophylactically or as adjuvant therapy in controlling haemorrhage in women undergoing treatment for Caesarean scar or cervical pregnancy. The catheter is introduced under ultrasonography guidance and is well tolerated. It has been shown to reduce complications and the need for surgical intervention.

### Miscellaneous

#### Assisted laparoscopic port wound closure^43^

Along with control of port site bleeding, use of a Foley catheter has been reported in the safe closure of laparoscopic port wounds, ensuring good fascial approximation. It was found that traction using the Foley balloon against the port site allows better visualisation of the fascial edges, thereby enabling easy and secure closure.

#### Totally extraperitoneal hernia repair^44^

The Foley catheter has been used to create preperitoneal space during laparoscopic extraperitoneal hernia repairs. It has been found to simplify the procedure and reduce operative time.

#### Blocking bronchial tubes (for one-lung deflation)^45^

A Foley catheter has been employed to assist one-lung anaesthesia in cases where double lumen bronchial tubes fail to function as desired. The Foley catheter is inserted through the tubes into one of the bronchi. The balloon is then inflated to block that bronchus, allowing the lung to deflate.

#### Cardiac surgery

Use of the Foley catheter has been described in cardiac surgery for a wide range of applications. These include:

•  as an endovascular clamp where standard vascular clamps are deemed appropriate either for occlusion of vessels or for control of bleeding;^46^
•  for the internal snaring of the inferior vena cava (IVC) and superior vena cava (SVC) in “redo” tricuspid surgery where it becomes challenging to dissect the IVC and SVC because of dense adhesions;^47^
•  preventing air embolism during mitral valve surgery, when the catheter may be used to vent the air bubbles;^46,47^
•  for endovascular occlusion of the aorta through the left ventricular apex in cases of porcelain vessels.^48^

#### Neonatal genital prolapse^49^

Neonatal genital prolapse is a rare condition associated almost invariably with other congenital malformations. Foley catheters have been used successfully in the conservative management of such cases. The catheter is introduced after manual reduction of the prolapse, and this helps in stabilising and maintaining the reduction.

#### Gaining access for retroperitoneoscopy^50^

Retroperitoneal laparoscopy is a procedure that provides direct access to the retroperitoneal organs and avoids the complications associated with transperitoneal laparoscopy. A glove finger technique has traditionally been employed to create a space in the dense retroperitoneal fat in order to gain access. However, a Foley catheter can also be used for this purpose. It is simple, effective and consistent. In addition, it has the added benefit of being directed into the required position accurately and consistently owing to the shape of its firm, conical tip, which the original glove finger technique could not provide.

## Discussion

Our main aim with this review article was to highlight the widespread applications of the Foley catheter, one of the most commonly used devices in modern surgical practice. With advances in technology and medical innovation, there is a real danger that the use of such a simple and inexpensive device could fall by the wayside. With this in mind, we decided to search the literature to identify and compile its various applications.

In this article, we have covered the Foley catheter’s history, structure and modifications since its invention as well as its various common and more rare applications in medical practice. The Foley catheter has come a long way from being a simple hollow metal tube to the silver impregnated, three-way silicone catheters of today. Along with these structural changes, it has also shown a wide range of functions. It has been used for drainage of urine and to control major haemorrhage. Some of the applications described above will be familiar to most surgeons whereas some are less well known. The range of applications covered demonstrates the versatile nature of this deceptively simple and humble device.

## Conclusions

It should be noted that this is not an exhaustive review of the uses of the Foley catheter; there are plenty of other applications not covered in this brief article. While the innovative thinking of practitioners in utilising a Foley catheter in novel and unconventional ways is worthy of recognition, the implications for practice (in terms of use as an unlicensed product, consent, ethics and professional responsibilities) must not be overlooked. Appropriate research and qualification of these novel applications before utilising them in clinical practice is therefore recommended.
